# Supplementary material for: Factors influencing the practice of Smoking Cessation Assessment and Management among Primary Care Doctors (SCAAM-DOC) in three districts of Malaysia
Source: PLoS One. 2022 Sep 29;17(9):e0274568. doi: 10.1371/journal.pone.0274568 (PMC9522281; doi:10.1371/journal.pone.0274568)

S1 File. Questionnaire for data collection

**Title:**

Factors influencing the practice of smoking cessation assessment and management among primary health care doctors in Selangor primary health care clinics (SCAAM-DOC)

**SECTION I: PERSONAL DETAILS**

| No | Details | Information |
| --- | --- | --- |
|  | Age (years): |  |
|  | Gender: |  |
|  | Years of service: |  |
|  | Occupation:  Option   1. Intern 2. Medical officer |  |
|  | Smoking status:  (Yes / No / Ex-smoker) |  |
|  | In the last 1 month, how many smokers have you encountered during your clinic consultation? |  |
|  | In the past 1 month, did you encounter any smoker during your clinic consultation? |  |
|  | How many of the smokers you have encountered during your clinic consultation in the past 1 month are were willing to quit smoking? |  |

**SECTION II – ORGANIZATION SUPPORT**

1. Do you think your clinic has been provided with enough of smoking cessation banners, brochures and leaflets (any of these)?

**Answer:** YES / NO

1. Are there any designated Quit Smoking Clinic in your center?

**Answer:** YES / NO

1. Have you attended any training or courses for smoking cessation?

**Answer:** YES / NO

If yes, when was the most recent training course?

_________________________

1. Are there availability of medications for nicotine replacement in your clinic?

**Answer:** YES / NO

**SECTION III – KNOWLEDGE, ATTITUDE AND PRACTICE IN SMOKING CESSATION**

(Please tick your answer)


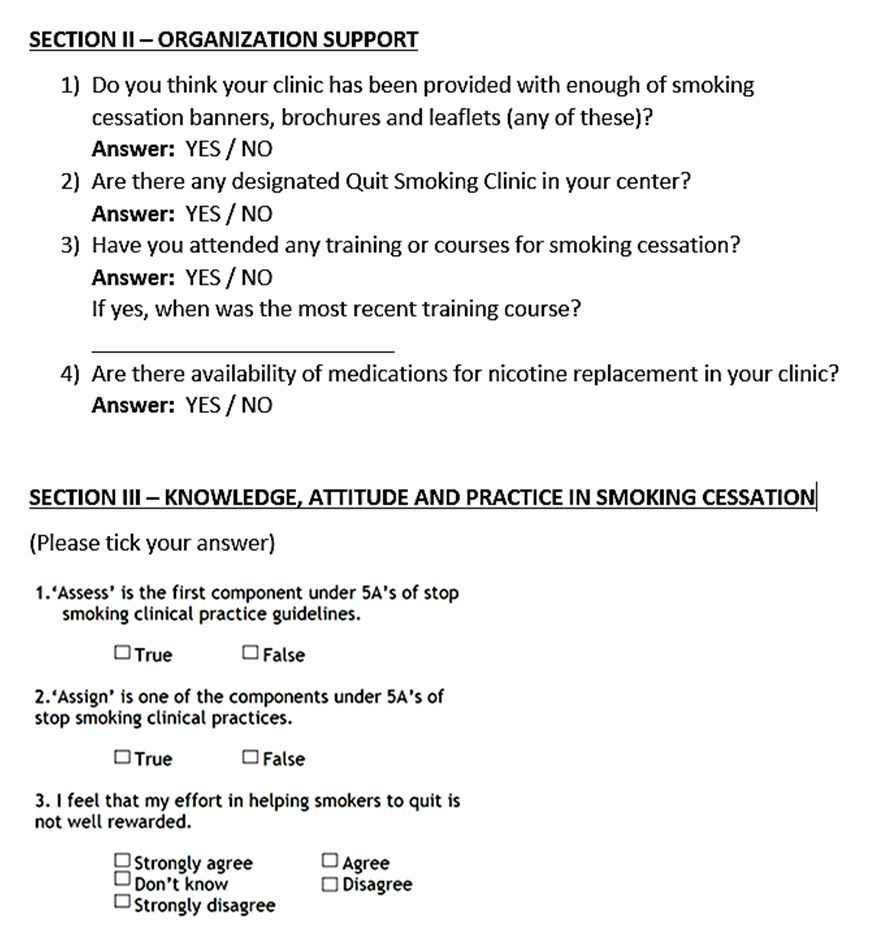


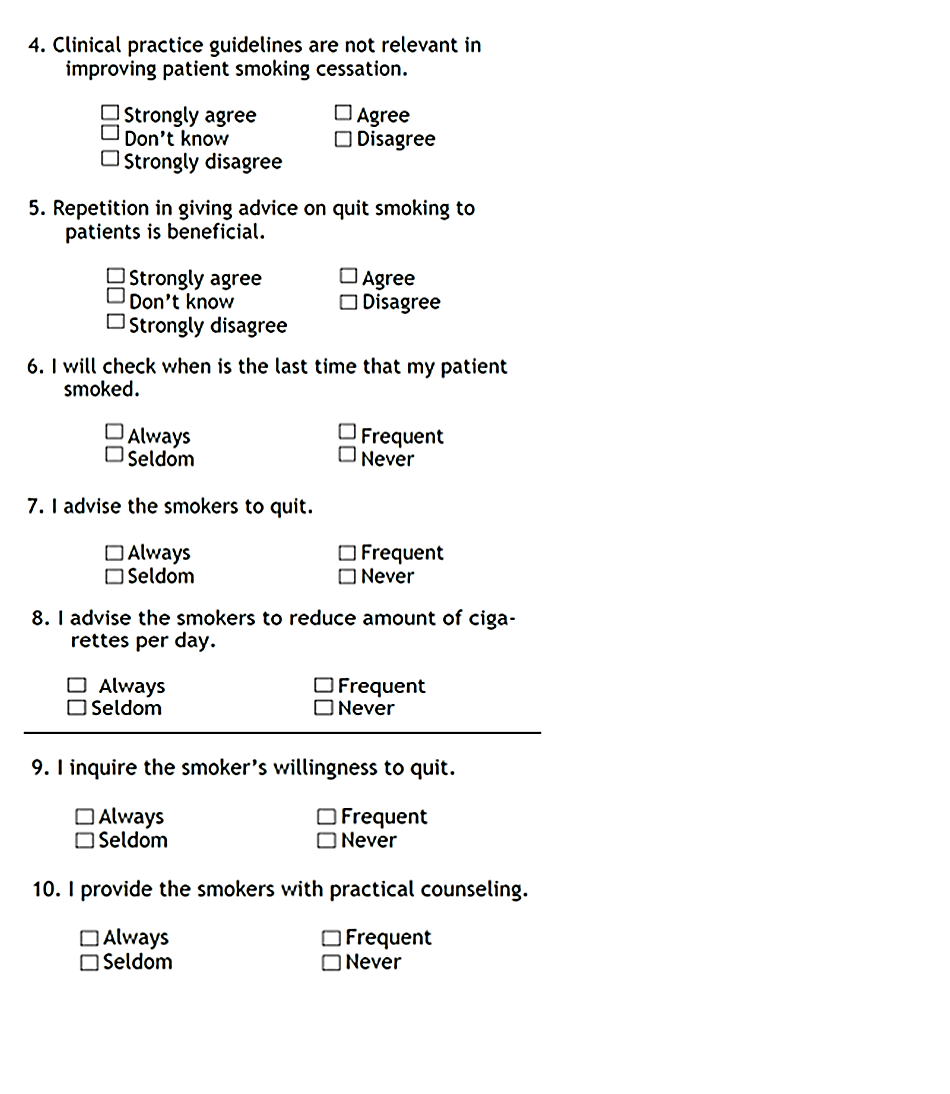


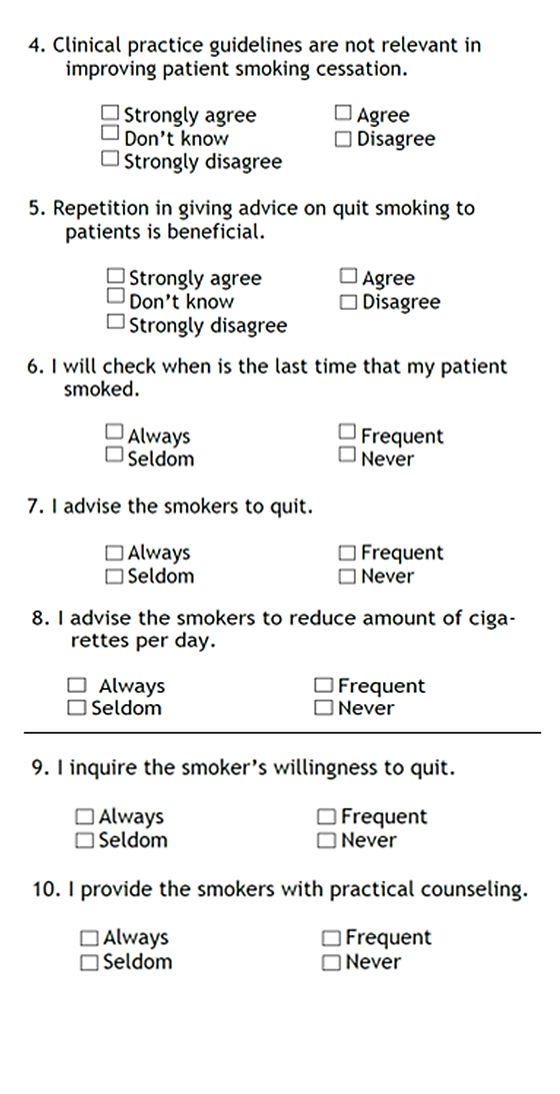


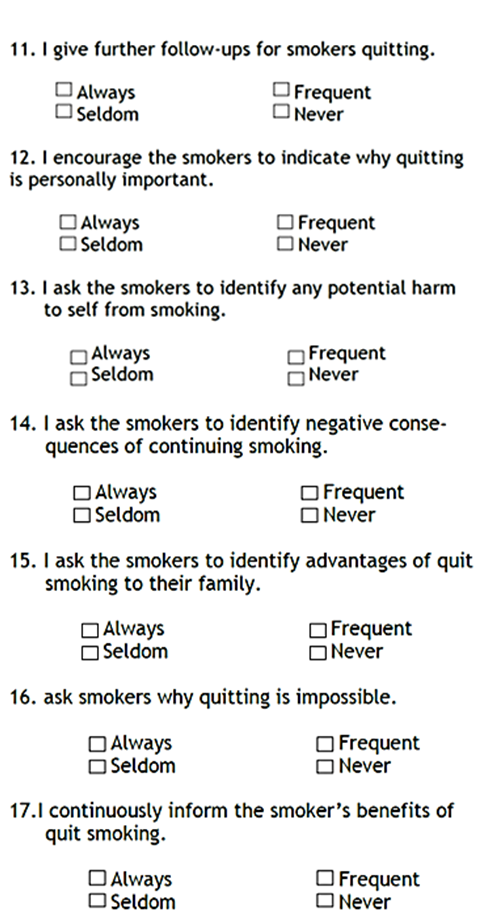

Supplement: S1 File — (DOCX) [file pone.0274568.s001.docx]
