# Supplementary material for: Factors influencing the practice of Smoking Cessation Assessment and Management among Primary Care Doctors (SCAAM-DOC) in three districts of Malaysia
Source: PLoS One. 2022 Sep 29;17(9):e0274568. doi: 10.1371/journal.pone.0274568 (PMC9522281; doi:10.1371/journal.pone.0274568)
Supplement: S2 File — (DOCX) [file pone.0274568.s002.docx]

S2 File. Factors associated with the practice of smoking cessation management at contemplation phase among primary health care doctors

|  | **Preliminary model**  **(Simple Logistic Regression)** | | | | **Final model**  **(Multiple Logistic Regression)** | | | |
| --- | --- | --- | --- | --- | --- | --- | --- | --- |
|  | **COR** | **95% CI** | | **p- value** | **AOR** | **95% CI** | | **p- value** |
|  |  | **Lower** | **Upper** |  |  | **Lower** | **Upper** |  |
| **Socio**  **Demographic** |  |  |  |  |  |  |  |  |
| **Factors:** |  |  |  |  |  |  |  |  |
| **Age** |  |  |  |  |  |  |  |  |
| ≥41 years | 1.00 |  |  |  | 1.00 |  |  |  |
| 36 to 40 years | 1.25 | 0.46 | 3.34 | 0.66 | 1.33 | 0.47 | 3.82 | 0.59 |
| 31 to 35 years | 1.75 | 0.73 | 4.16 | **0.21** | 1.98 | 0.61 | 6.91 | 0.24 |
| ≤30 years | 2.12 | 0.81 | 5.57 | **0.13** | 3.46 | 0.80 | 14.97 | 0.10 |
| **Gender** |  |  |  |  |  |  |  |  |
| Female | 1.00 |  |  |  |  |  |  |  |
| Male | 0.78 | 0.46 | 1.33 | 0.36 |  |  |  |  |
| **Years**  **of service** |  |  |  |  |  |  |  |  |
| ≥11 years | 1.00 |  |  |  | 1.00 |  |  |  |
| 6 to 10 years | 1.52 | 0.83 | 2.79 | **0.17** | 0.94 | 0.38 | 2.35 | 0.90 |
| ≤5 years | 1.37 | 0.69 | 2.72 | **0.37** | 0.54 | 0.17 | 1.76 | 0.31 |
| **Occupation** |  |  |  |  |  |  |  |  |
| Medical officer | 1.00 |  |  |  |  |  |  |  |
| Intern | 1.61 | 0.19 | 13.99 | 0.66 |  |  |  |  |
| **Smoking status** |  |  |  |  |  |  |  |  |
| Never smoker | 1.00 |  |  |  |  |  |  |  |
| Former smoker | 1.28 | 0.27 | 6.14 | 0.76 |  |  |  |  |
| Current smoker | 0.32 | 0.02 | 5.17 | 0.42 |  |  |  |  |
| **Organization support:** |  |  |  |  |  |  |  |  |
| **Smoking cessation banners, brochures and leaflets** |  |  |  |  |  |  |  |  |
| Yes | 1.00 |  |  |  |  |  |  |  |
| No | 1.26 | 0.78 | 2.03 | 0.35 |  |  |  |  |
| **Designated smoking cessation clinic** |  |  |  |  |  |  |  |  |
| Yes | 1.00 |  |  |  |  |  |  |  |
| No | 0.74 | 0.40 | 1.37 | 0.33 |  |  |  |  |
| **Training courses for smoking cessation** |  |  |  |  |  |  |  |  |
| Yes | 1.00 |  |  |  |  |  |  |  |
| No | 0.87 | 0.44 | 1.73 | 0.69 |  |  |  |  |
| **Nicotine replacement medication** |  |  |  |  |  |  |  |  |
| Yes | 1.00 |  |  |  |  |  |  |  |
| No | 1.05 | 0.60 | 1.84 | 0.86 |  |  |  |  |
| **Knowledge**  **(K) Score** |  |  |  |  |  |  |  |  |
| Good=2 | 1.00 |  |  |  | 1.00 |  |  |  |
| Average=1 | 1.09 | 0.53 | 2.24 | 0.82 | 1.02 | 0.49 | 2.13 | 0.96 |
| Poor=0 | 1.59 | 0.86 | 2.96 | **0.14** | 1.50 | 0.79 | 2.85 | 0.22 |
| **Attitude(A)** |  |  |  |  |  |  |  |  |
| **Median score** |  |  |  |  |  |  |  |  |
| Good>2 | 1.00 |  |  |  |  |  |  |  |
| Poor ≤2 | 1.00 | 0.62 | 1.60 | 0.99 |  |  |  |  |

COR crude odds ratio

AOR adjusted odds ratio
